# Spectrum of care approach to animal shelter management of feline infectious peritonitis complicated by feline leukemia virus

**DOI:** 10.3389/fvets.2025.1570267

**Published:** 2025-09-29

**Authors:** Emma K. LaVigne, Nicole E. Levy, Alexis R. Bardzinski, Federica Scaletti, Kevin Horecka, Mia K. Cuccinello, Dana M. Moore, Julie K. Levy

**Affiliations:** ^1^Small Animal Clinical Sciences, University of Florida, Gainesville, FL, United States; ^2^Austin Pets Alive!, Austin, TX, United States; ^3^TheraPet Research and Consulting LLC, Austin, TX, United States; ^4^Research Department, American Pets Alive!, Austin, TX, United States; ^5^Shelter Medicine Program, University of Florida, Gainesville, FL, United States

**Keywords:** animal shelter, cats, FIP, feline infectious peritonitis, FeLV, feline leukemia virus, GS-441524, spectrum of care

## Abstract

**Introduction:**

Feline infectious peritonitis (FIP) occurs most commonly in multi-cat environments such as animal shelters. FIP is often suspected based on compatible history, signalment, physical examination, and hematological and biochemical findings. Increased diagnostic certainty requires additional diagnostic imaging and laboratory testing that may be out of reach for resource-poor caregivers and organizations. The objective of this retrospective study was to evaluate response to GS-441524 (GS) therapy of cats diagnosed with FIP using a spectrum of care approach relying on physical examination, hematology, biochemistry, and Feline Leukemia Virus (FeLV) antigen at a shelter specializing in the adoption of cats with FeLV.

**Methods:**

The FIP treatment protocol included treatment with GS for 84 days, followed by observation for 84 days. Starting dosage, dose adjustment, route of administration, and treatment extensions were based on initial condition and response to therapy. Hematology and biochemistry panels were performed at baseline, treatment, and post-treatment observation periods. Response to treatment and survival times were compared between FeLV-positive and FeLV-negative cats.

**Results:**

A total of 170 cats diagnosed with FIP (104 FeLV-positive; 66 FeLV-negative) were included in the study. Hematology and serum chemistry abnormalities and their resolution during treatment were not significantly different between the groups, nor were there significant differences in survival through the treatment period (79% vs. 76%) or observation period (74% vs. 74%) between FeLV-positive and FeLV-negative cats respectively. Most mortality (55%) occurred in the first 7 days; cats surviving >7 days had an 86% survival rate. Despite equivalent responses to GS treatment, the FeLV-positive group experienced progressive mortality following the observation period. Survival of FeLV-positive cats (median 524 days, range 1-1585 days) was significantly shorter than for FeLV-negative cats (median not reached, range 0-1424 days) (*p* = 0.0001). Fifteen cats (11%) that achieved remission of FIP during the initial treatment experienced one or more episodes of FIP relapse up to 1.2 years later (11 FeLV-positive; 4 FeLV-negative).

**Discussion:**

The spectrum of care diagnostic approach appeared to be sufficient and preserved resources for the successful treatment of shelter cats. FeLV-positive cats achieved equivalent FIP remission rates to FeLV-negative cats but were still at risk for shortened lifespan associated with FeLV infection.

## 1 Introduction

Feline enteric coronavirus (FCoV) is a globally distributed highly infectious but usually asymptomatic virus spread by the fecal-oral route among cats. It has especially high prevalence and viral replication rates in multi-cat environments such as animal shelters and breeding catteries (1, 2). In a small proportion of FCoV-infected cats, viral mutations and host susceptibility factors result in feline infectious peritonitis (FIP), a systemic inflammatory syndrome that typically results in death within weeks of FIP diagnosis.

FIP was considered untreatable until the discovery that the antiviral compound GS-441524 (GS), a parent nucleoside of GS-5734 (Remdesivir), was effective against FIP (3, 4). This triggered a surge in research and treatment with GS and related compounds with reported survival rates in naturally infected cats ranging from 55 to 100% (3–19). High treatment success rates along with a lack of commercially available GS spawned social networks of cat owners who gathered data, created treatment protocols, and helped tens of thousands of owners seek international sources of GS to save their cats. Veterinarians could prescribe GS in Australia, the UK, and parts of Europe for several years before it became widely available from compounding pharmacies in the US in 2024.

Feline leukemia virus (FeLV), another globally distributed virus of cats, is associated with bone marrow suppression, lymphosarcoma, and immune dysfunction impairing responses to other infectious diseases (34). In one study of 652 FeLV-positive cats at the shelter in this report, mortality prior to adoption was 17%, and of those, 61% of deaths were suspected to be related to co-infection with FIP (20). FIP was the most common infectious disease diagnosed at the time of necropsy in 396 FeLV-positive cats (21). FeLV decreases average lifespan in infected cats, particularly when a high FeLV proviral load is present at the time of diagnosis (22–26).

The animal shelter in this study applied a spectrum of care approach based on the AAVMC Spectrum of Care Initiative (27). The spectrum of care approach allows for a “range of flexible diagnostic and treatment options that a veterinarian can provide based on various factors such as financial resources, veterinarian and practice abilities, and client goals, while also adhering to evidence-based medicine. It takes into consideration scientific evidence, veterinarian expertise, and individual circumstances of clients and patients, while acknowledging that there is not a single standard of care applicable to every case” (27). Currently there is no “gold standard” diagnostic test for FIP, so veterinarians integrate a combination of compatible presenting clinical signs, physical exam findings, and clinicopathologic data.

Current guidelines for high-confidence confirmation of FIP diagnosis include a multilayered approach beginning with history, compatible clinical signs, physical examination findings, and routine hematological and serum biochemical analysis. If effusions are present, they may be assessed by cytology, biochemistry, immunocytochemistry, and RT-PCR. Diagnostic imaging may include radiography, ultrasonography, and cross-sectional imaging. More invasive testing may include cerebrospinal fluid or aqueous humor analysis, tissue aspiration, or exploratory surgery and tissue biopsy. While comprehensive testing may increase the confidence of diagnosis, it may be unavailable to resource-scarce facilities, delay treatment, increase risk of adverse events, and increase the cost of diagnosis beyond the cost of treatment itself. The European Advisory Board on Cat Diseases (ABCD) provides an FIP Diagnostic Tool that includes an option for a treatment trial when there is a high index of suspicion for FIP, stating that “A rapid and sustained positive response to antiviral treatment is a means of supporting a diagnosis of FIP” (1).

While GS is highly effective in the treatment of cats with FIP, treatment outcomes in a large cohort of shelter cats, including those co-infected with the immunosuppressive retrovirus FeLV have not been reported (28). The purpose of this retrospective study was to evaluate the response of cats to treatment with GS following a spectrum of care approach to diagnosis of FIP relying on physical examination, routine hematological and biochemical testing, and point-of-care testing for FeLV antigen. The aforementioned were used to both establish a clinical diagnosis of FIP (possibly complicated by co-infection with FeLV) and to provide a baseline against which to monitor clinicopathological normalization in response to therapy.

## 2 Materials and methods

### 2.1 Animals

#### 2.1.1 Shelter facility

The shelter in this study, Austin Pets Alive! (APA!), is a non-profit animal shelter focused primarily on transferring cats and dogs at risk for euthanasia from other regional shelters to their facilities or foster programs in Austin, Texas. APA! has an FeLV adoption program as well as a large FIP treatment program. The shelter has an in-house veterinary clinic, and euthanasia of cats was reserved for those deemed to be untreatable with irremediable suffering. All cats received standard preventive healthcare, including core vaccines and parasiticides at the time of admission to the shelter and were treated for medical conditions as needed in the shelter clinic as previously described (20). Cats were tested for FeLV using anticoagulated whole blood in a point-of-care p27 antigen test with reported sensitivity of 100% and specificity of 98–100% (SNAP FeLV, IDEXX Laboratories, Westbrook Maine, USA) (29, 30). In accordance with the shelter’s spectrum of care approach, the results of the FeLV antigen test were used to classify the FeLV status of the cats without PCR confirmation with the understanding that some cats might be misclassified as false-positives or false-negatives.

#### 2.1.2 Study inclusion and exclusion criteria

Shelter-owned cats diagnosed with FIP by the shelter veterinarians and treated with GS in volunteer foster homes from September 2020 to August 2023 were included in the study. Electronic medical records were reviewed for retrospective collection of patient data. To reduce confounding factors, cats were excluded if they were diagnosed with major comorbidities at the start of treatment, such as neoplasia, diabetes, congestive heart failure, pneumonia, stomatitis, or other infectious diseases. Cats receiving more than 10% of GS doses from alternative sources were also excluded. This occasionally occurred if GS was started at another facility prior to transfer to the APA! shelter.

### 2.2 FIP diagnosis

In this shelter, veterinarians developed a resource-sparing spectrum of care diagnostic protocol based on compatible clinical signs, physical exam findings, and hematological and serum biochemical results. Compatible clinical signs ranged from a combination of generally nonspecific findings of anorexia, weight loss, muscle atrophy, fever, dyspnea, or icterus to signs of greater suspicion, including uveitis, neurological signs, and abdominal and/or thoracic effusion. Laboratory values supporting a diagnosis of FIP in cats with compatible clinical signs included decreased hematocrit, hemoglobin, lymphocytes, albumin, or albumin to globulin ratio and increased globulin, total protein, or neutrophils (1, 2, 31). Diagnostic imaging, effusion analysis, PCR, and histopathology were not routinely performed in the spectrum of care protocol.

### 2.3 FIP treatment

#### 2.3.1 Treatment protocol

Cats were managed as outpatients and lived with their volunteer foster caregivers who administered the GS medication. GS (Natural Micron Pharm Tech; Tai’an, China) was analyzed by HPLC to confirm composition and purity (>99%) and by UV spectrophotometry to confirm concentration. GS was compounded (Bloom Biosciences, Austin, Texas, USA) in two injectable concentrations (17.5 mg/mL or 20 mg/mL), and oral capsules in 3 strengths (10 mg, 20 mg, and 40 mg). Cats were initially treated with injectable medication to assure absorption and full dose delivery according to Table 1. As their condition stabilized, most were transitioned to oral medication at the discretion of the attending veterinarian. Cats in stable condition with non-effusive FIP were treated once daily, and cats in all other conditions were started with twice daily medication and transitioned to daily treatment after stabilization (approximately 5 days). Treatment was continued for 84 days with extensions for cats that had not achieved remission by that time. The dosage was increased to 15–20 mg/kg if poor response to treatment was noted within 2 weeks. After 2–4 weeks of injectable treatment, cats were transitioned to oral capsules at a dosage of twice the injectable due to lack of information regarding oral bioavailability in 2020 when the shelter began treating cats for FIP.

**Table 1:** Starting dose chart for GS-441524 treatment as a function for FeLV status and clinical presentation.

FIP Type	FeLV-positive	FeLV-negative
Non-effusive	12 mg/kg SC q 24 h	10 mg/kg SC q 24 h
Effusive	10 mg/kg SC q 12 h	10 mg/kg SC q 12 h
Neurological/Ocular	12 mg/kg SC q 12 h	10 mg/kg SC q 12 h
Critically ill	15–30 mg/kg SC q 12 h	12–30 mg/kg SC q 12 h

#### 2.3.2 Monitoring and follow-up

Cats underwent physical examination, CBC, and serum chemistry evaluation by the shelter veterinarians approximately every 4 weeks. Treatment was extended if any evidence of FIP remained. If clinical signs and laboratory results were normalized at the end of the treatment period, cats were subsequently placed in a 12-week observation period, after which they were considered in remission/cured if they remained free of evidence of FIP. Cats that appeared healthy after completing treatment, but subsequently developed recurrent clinical signs or laboratory findings consistent with FIP were considered relapsed. Cats with relapse of FIP were retreated with a higher dose of GS (15–30 mg/kg). Following the observation period, cat adopters were contacted approximately every 3 months for status updates. Follow-up was continued through July 2025, allowing a minimum of 1-year follow-up for all cats in the study.

### 2.4 Statistical analysis

#### 2.4.1 Data analysis

All statistical analyses were performed using Python (version 3.12) with scipy, scikit-learn, and pandas libraries. A two-tailed significance level of *α* = 0.05 was used for all statistical tests.

#### 2.4.2 Descriptive statistics and group comparisons

Sex and FIP type at the time of diagnosis and chemistry and hematology values at each time point were compared between FeLV-positive and FeLV-negative groups using Chi-squared or Fisher’s exact tests as appropriate. The age distribution between FeLV-positive and FeLV-negative groups was compared using independent t-tests and non-parametric Mann–Whitney U tests.

#### 2.4.3 Survival analysis

Kaplan–Meier survival curves were generated and compared using a log-rank test. Survival to the end of the observation period was analyzed as a binary outcome (survived vs. did not survive) using both parametric and non-parametric approaches. A full multivariable logistic regression model (parametric analysis) was constructed including FeLV status (positive/negative), age at diagnosis (continuous), and FIP type (effusive/non-effusive/ocular-neuro) as predictor variables. The model achieved an accuracy of 75% in predicting survival outcomes. Non-parametric analyses (Spearman Correlations and Kruskal-Wallis Tests) were performed to assess the relationship between variables and survival without assuming normal distributions.

#### 2.4.4 Correlation analyses

Pearson correlation analyses were performed to assess the relationship between continuous variables and survival through the observation period.

#### 2.4.5 Effect size calculations

Odds ratios were calculated for categorical variables.

## 3 Results

### 3.1 Animals, age, type of FIP

**Figure 1:**
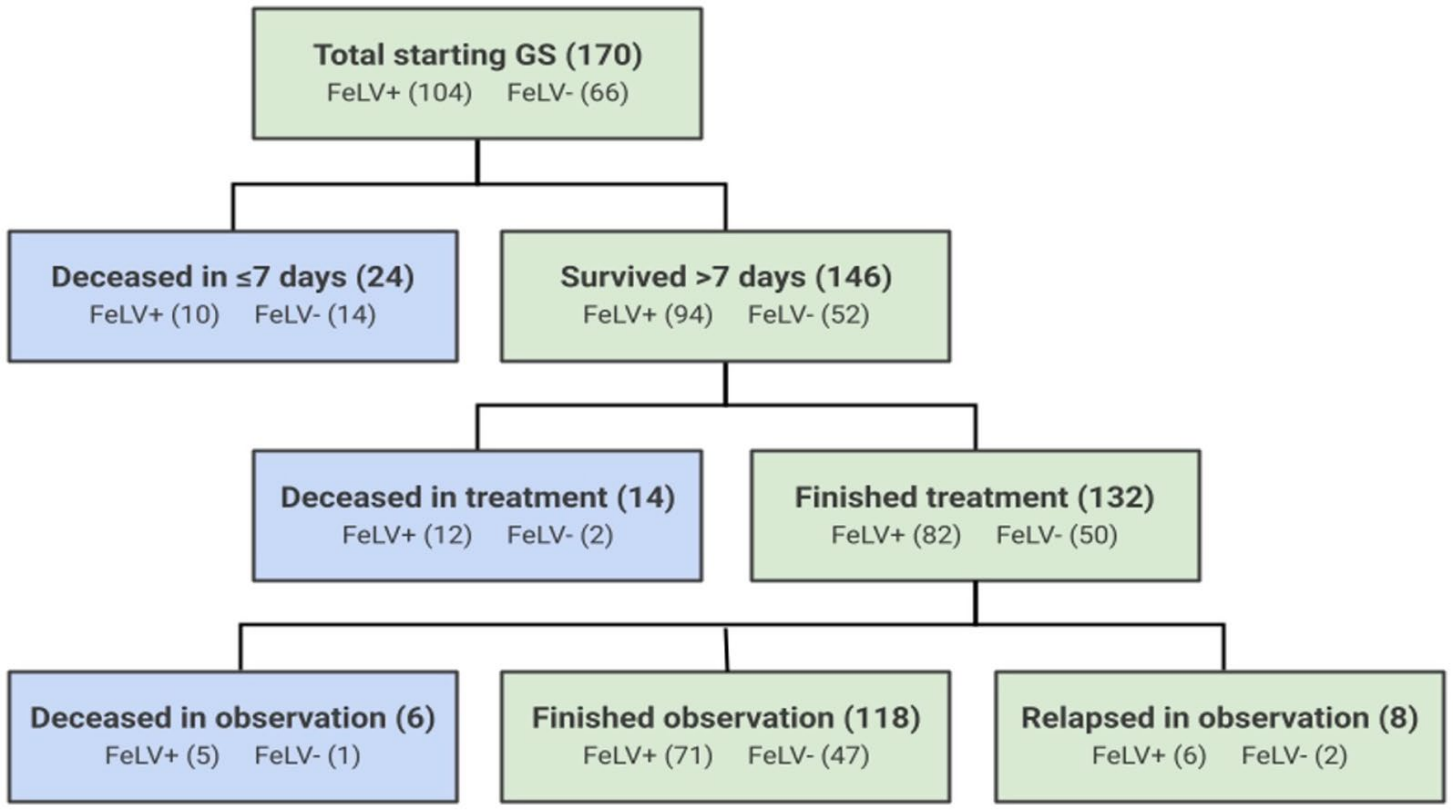
Outcomes of 170 cats treated with GS-441524 for FIP. Blue boxes represent cats that died or were euthanized during the treatment or observation periods, and green boxes represent surviving cats.

A total of 170 cats diagnosed with FIP met the study enrollment criteria. Of these, 104 were categorized as FeLV-positive and 66 were categorized as FeLV-negative based on FeLV antigen test results prior to therapy (Figure 1). Source of cats, their sex and age at time of diagnosis, and clinical FIP presentation type are described in Table 2. FeLV-negative cats were more likely to be transferred from other organizations with pre-existing FIP (*p* = 0.0001), and FeLV-positive cats were more likely to be residing in the shelter at the time of diagnosis (*p* = 0.01). FeLV-positive cats were significantly older at the time of diagnosis than FeLV-negative cats (mean age: 6.2 ± 6.0 months vs. 3.1 ± 3.6 months, respectively; *p* < 0.001). This age difference of 3.1 months represented a medium effect size (Cohen’s d = 0.6). FeLV-positive cats were also older on average (median 11.7 months, range 1.0–121.5) than FeLV-negative cats (median 4.1 months, range 0–60.1) at the time of shelter admission (*p* < 0.0001).

**Table 2:** Source, signalment, and FIP type at time of diagnosis by FeLV status at the start of treatment and in cats surviving through the observation period.

Characteristic		Total enrolled cats			Cats surviving through observation		
		FeLV+ (*n* = 104)	FeLV− (*n* = 66)	*p*-value	FeLV+ (*n* = 77)	FeLV− (*n* = 49)	*p*-value
Source	Transfer-in with FIP	14 (13%)	26 (39%)	**0.0001**	12/14 (86%)	22/26 (85%)	1.0
	APA! In Shelter FIP	53 (51%)	21 (32%)	**0.01**	38/53 (72%)	13/21 (62%)	0.4
	Post-adoption FIP	37 (36%)	19 (29%)	0.4	27/37 (73%)	14/19 (74%)	1.0
Sex	Male neutered	53 (51%)	28 (42%)	0.3	37/53 (70%)	23/28 (82%)	0.3
	Male intact	9 (9%)	10 (15%)	0.2	5/9 (56%)	6/10 (60%)	1.0
	Female spayed	33 (32%)	15 (23%)	0.2	26/33 (79%)	11/15 (73%)	0.7
	Female intact	9 (9%)	13 (20%)	**0.04**	9/9 (100%)	9/13 (69%)	0.1
Age	0–6 months	21 (20%)	36 (55%)	**<0.0001**	19/21 (90%)	28/36 (78%)	0.3
	7–11 months	22 (21%)	12 (18%)	0.6	17/22 (77%)	10/12 (83%)	1.0
	1–2 years	33 (32%)	11 (17%)	**0.03**	26/33 (79%)	6/11 (55%)	0.1
	3–5 years	21 (20%)	7 (11%)	0.10	12/21 (57%)	5/7 (71%)	0.7
	>5 years	7 (7%)	0 (0%)	**0.04**	3/7 (43%)	N/A	N/A
Type of FIP	Effusive	40 (38%)	24 (36%)	0.8	33/40 (80%)	18/24 (75%)	0.5
	Non-effusive	26 (25%)	7 (11%)	**0.02**	17/26 (65%)	4/7 (57%)	0.7
	Ocular and/or neurological	38 (37%)	35 (53%)	**0.03**	27/38 (71%)	27/35 (77%)	0.6

Bold values indicate statistical significance.

### 3.2 Treatments

A total of 132 cats (78%) completed treatment, including 82 (62%) receiving a standard 84 ± 5 day course of GS (Table 3). Fifty cats (38%) had treatment extensions due to various reasons including spay/neuter, persistent physical or diagnostic testing abnormalities, or caregiver error (median 18 days; range 6–101). A total of 31 cats had dose escalations after failing to respond to the initial dose as expected (Table 3). There was no significant difference in survival through observation between FeLV-positive and FeLV-negative cats that had a normal treatment length, treatment extension, dose escalation, or mortality during treatment.

**Table 3:** FIP treatment variations and survival in cats with and without FeLV.

Treatment	Total enrolled cats			Cats surviving through observation		
	FeLV+ (*n* = 102*)	FeLV− (*n* = 66)	*p*-value	FeLV+ (*n* = 75*)	FeLV− (*n* = 49)	*p*-value
Standard treatment	51 (50%)	31 (47%)	0.7	50/51 (98%)	30/31 (97%)	1.0
Extended treatment duration	31 (30%)	19 (29%)	0.8	27/31 (87%)	19/19 (100%)	0.3
Dose escalation during initial treatment	17 (17%)	14 (21%)	0.5	13/17 (76%)	11/14 (79%)	1.0
Mortality during treatment	22 (22%)	16 (24%)	0.7	N/A	N/A	N/A

*Two cats had undocumented treatment lengths.

### 3.3 Laboratory results

**Figure 2:**
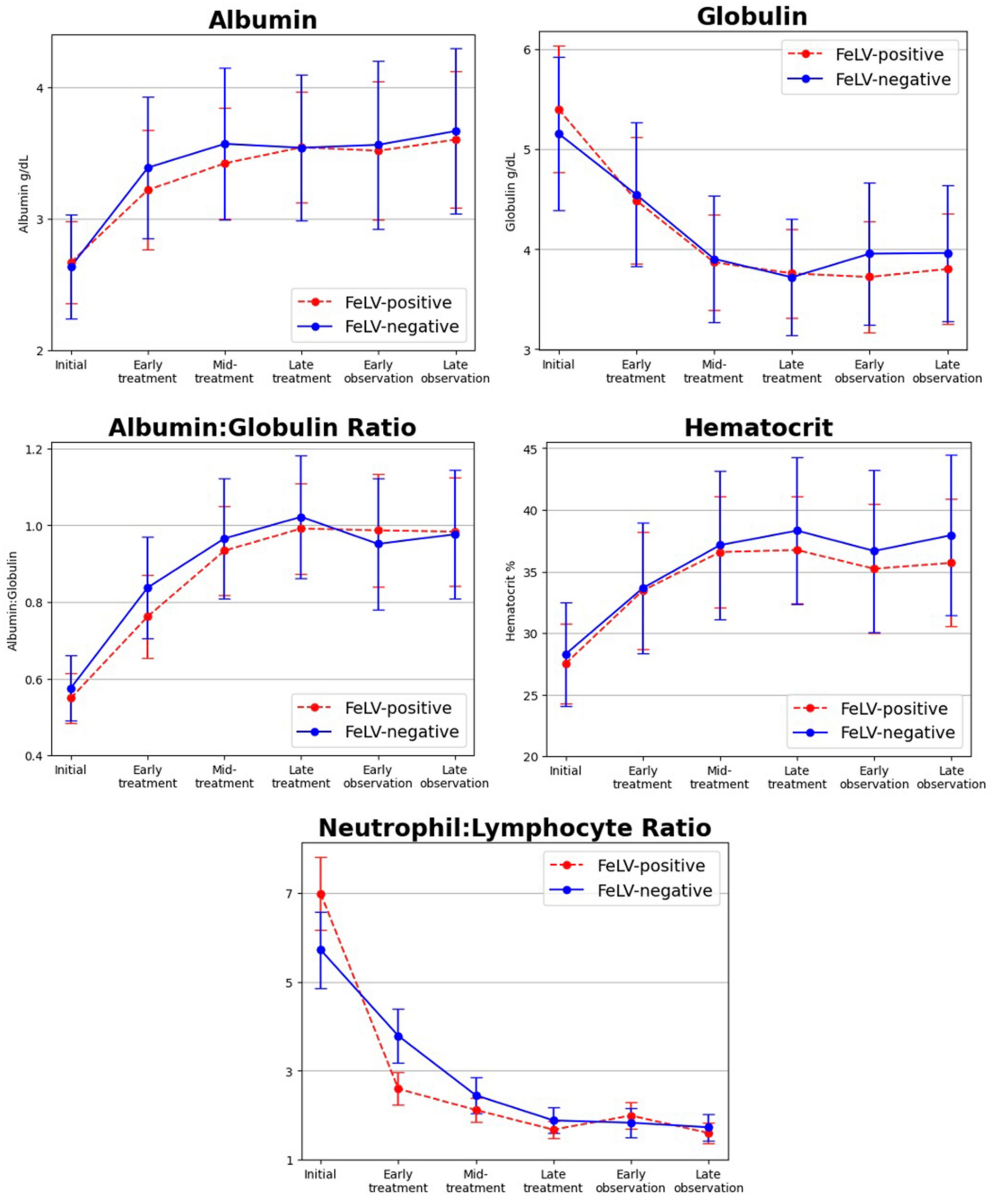
Changes in select bloodwork values over time from initial diagnosis and time points during treatment of FIP with GS-441524 and the post-treatment observation period. Albumin, albumin to globulin ratio, and hematocrit increased during treatment, whereas globulin and neutrophil to lymphocyte ratio decreased. Initial laboratory abnormalities and their normalization in response to treatment were not significantly different between FeLV+ (red dashed line) and FeLV− (blue solid line) cats.

Hematological and biochemical testing results mirrored previously reported findings in diagnosis of FIP and response to treatment (4–6, 8, 13, 14, 18, 19). In particular, hypoalbuminemia (44%), hyperglobulinemia (30%), low albumin to globulin ratio (41%), anemia (53%), lymphopenia (14%), neutrophilia (23%), and leukocytosis (27%) were common and resolved with treatment (Figure 2). Two outlier results were excluded from the initial time point for having neutrophil:lymphocyte ratio values > 80 (> 20 standard deviations from the mean) as they created the illusion of significant differences between FeLV-positive and FeLV-negative groups for that ratio. There were no significant differences in laboratory abnormalities or their resolution with treatment between FeLV-positive and FeLV-negative cats (*p* > 0.05; specifically, |t| < 0.4 and *p* > 0.4 for all tests even without multiple comparison correction).

### 3.4 Survival

**Figure 3:**
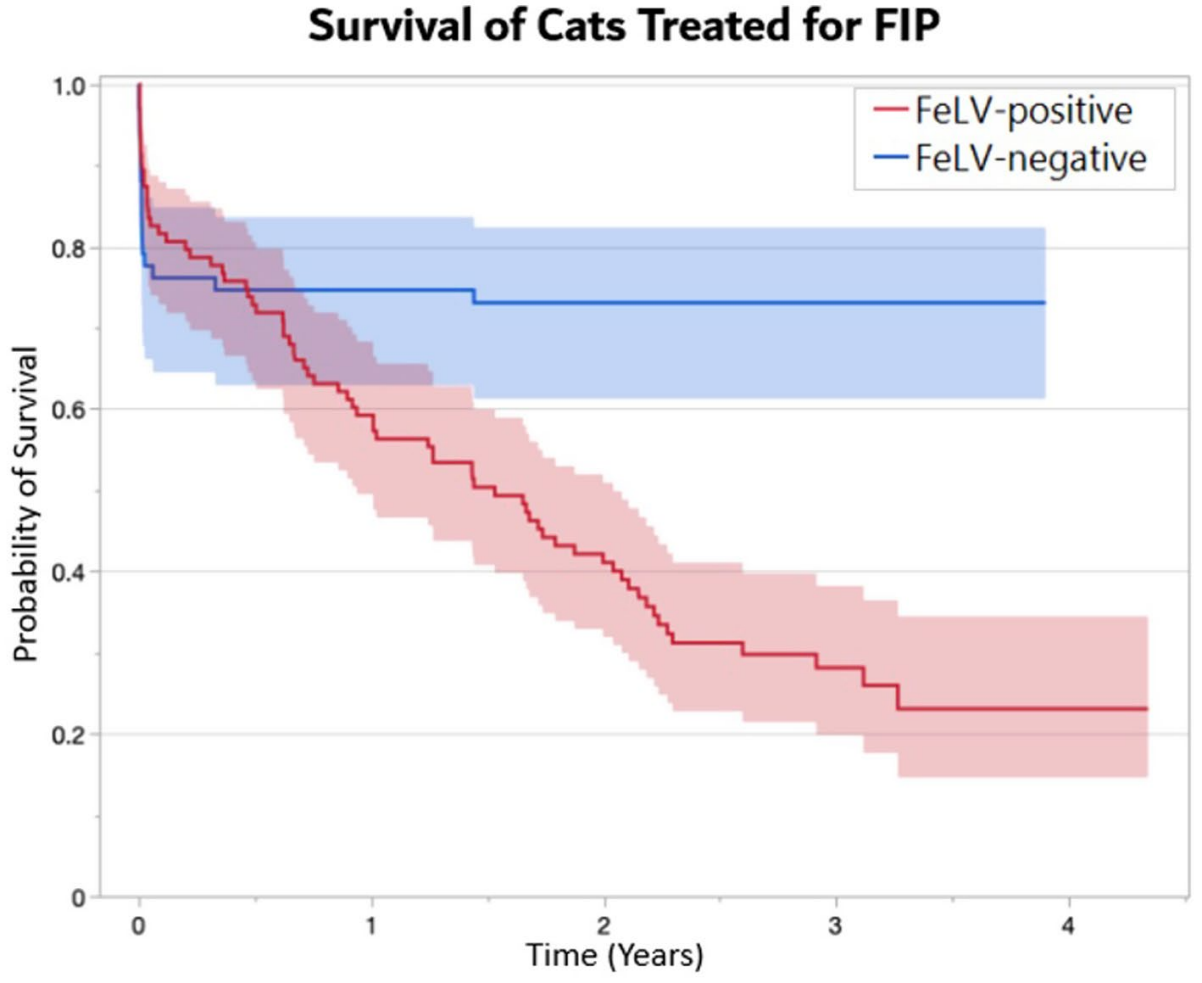
Kaplan-Meier survival curves demonstrating the probability of survival (± 95% CI) of FeLV-positive (*n* = 104) and FeLV-negative (*n* = 66) cats treated with GS-441524 for FIP. A median survival time of 1.5 years was documented in FeLV-positive cats, whereas median survival was not reached in FeLV-negative cats (*p* < 0.0001).

The outcome of cats was tracked for a minimum of 1 year and up to 4 years following the first day of treatment. One FeLV-negative cat was lost to follow up after 273 days. Overall, 55% of mortality occurred during the first 7 days of initial treatment (24 of 44 deaths) (Figure 1). Ninety percent of cats surviving greater than 7 days completed treatment (132 of 146), and 86% of cats surviving greater than 7 days completed the subsequent observation period (126 of 132). FeLV-positive cats were more likely to survive through the first 7 days of treatment (*p* = 0.03), but there was no difference in survival through the end of treatment between the FeLV-positive and FeLV-negative cats. A total of 74% of cats in both groups survived through the observation period (*p* = 1.0). All FeLV-negative cats surviving through observation were still alive at the one-year mark (except for the unknown status of the one that was lost to follow-up) (Table 4). In contrast, survival of the FeLV-positive cats progressively declined after the observation period. Survival of FeLV-positive cats (median 524 days, range 1–1,585 days) was significantly shorter than for FeLV-negative cats (median not reached; range 0–1,424) (*p* < 0.0001) (Figure 3).

**Table 4:** Survival rates at milestone time points for FeLV-positive and FeLV-negative cats.

Survival milestone	FeLV+ (*n* = 104)	FeLV− (*n* = 66)	*p*-value
Survived first week of treatment	94 (90%)	52 (79%)	**0.03**
Survived through initial treatment	82 (79%)	50 (76%)	0.6
Survived through initial observation	77 (74%)	49 (74%)	1.0
Survived first year after starting treatment	62 (60%)	48* (73%)	0.08
Relapsed after initial treatment	11 (13%)	4 (8%)	0.3

*One FeLV-negative cat lost to follow-up after 273 days. Bold values indicate statistical significance.

FeLV-positive cats that were younger at the time of diagnosis were more likely to survive through the observation period than FeLV-positive cats that were older at the time of diagnosis (*p* < 0.05, *R*^2^ = 0.7). This was found to be a linear effect. For every month older an FeLV-positive cat was at diagnosis, their odds of death increased about 8.5%, and every year about 70%. There was no association between age and survival in FeLV-negative cats (*p* > 0.5, *R*^2^ = 0.01). FeLV-positive cats more often had non-effusive FIP (*p* = 0.02), whereas FeLV-negative cats more often had ocular and/or neurological forms (*p* = 0.03). There were no differences in survival outcomes at the end of observation of cats based on source, sex, age, or FIP type between FeLV-positive and FeLV-negative cats.

Multivariable logistic regression identified age at diagnosis was a significant predictor of survival through the observation period with younger cats showing improved survival (*β* = −0.02, *p* < 0.001), but FeLV status (*β* = 0.2, *p* = 1.0) and FIP type (*β* = −0.1, *p* = 0.7) were not. Non-parametric analysis identified age at diagnosis versus survival was significant (*r* = −0.2, *p* = 0.01), but FeLV status versus survival (*r* = −0.002, *p* = 1.0) and FIP type vs. survival (*r* = −0.04, *p* = 0.6) were not. Spearman’s correlation performed for the FeLV-positive group separately (*n* = 104) showed that age at diagnosis versus survival was significant (*r* = −0.3, *p* = 0.01) and the Kruskal-Wallis test (age quartiles) was also significant (*H* = 8.0, *p* = 0.05). Spearman’s correlation performed for the FeLV-negative group separately (*n* = 66) showed that age at diagnosis versus survival was not significant (*r* = −0.1, *p* = 0.5) and the Kruskal-Wallis test (age quartiles) was also not significant (*H* = 7, *p* = 0.07). FeLV status Odds Ratio was 1.0 (95% confidence interval not calculated).

In summary, FeLV-positive cats were significantly older than FeLV-negative cats (*p* < 0.001), with a difference of 3.1 months, indicating age as a potential confounding variable. While parametric logistic regression showed age as significant in both groups (*p* < 0.001), non-parametric analysis revealed a key difference: In the FeLV-positive group, age significantly predicted survival through the observation period (Spearman *r* = −0.3, *p* = 0.01), whereas in the FeLV-negative group, age did not significantly predict survival (Spearman *r* = −0.1, *p* = 0.5). When controlling for age, FeLV status was not significantly associated with survival through observation in parametric models (*p* = 1.0). FIP type was not significantly associated with survival in any model (*p* > 0.05 for all analyses).

### 3.5 FIP relapse

A relapse of FIP was defined as recurrence of clinical signs following completion of the treatment period. Fifteen of 132 cats (11%) that survived the initial treatment period were diagnosed with a relapse of FIP (Table 4). This included 11 of 82 surviving FeLV-positive cats (13%) and 4 of 50 surviving FeLV-negative cats (8%), which were not significantly different (*p* = 0.3) (Table 4). The median time from the last day of treatment to diagnosis of first relapse was 59 days in the FeLV-positive cats (5–422 days) and 86 days in the FeLV-negative cats (range 3–448 days). All four relapsing FeLV-negative cats were diagnosed with neurological or ocular forms of FIP (100%) at the time of study enrollment, compared to only 3 of 11 relapsing FeLV-positive cats (27%). Two cats were diagnosed with a second relapse and one with a third relapse. All relapses were retreated with higher doses of GS. Six cats treated for relapse were still alive at the time of publication (Table 5).

**Table 5:** Characteristics of 15 cats at the time of study enrollment for the original episode of FIP and response to treatment of FIP relapse.

FeLV Status (Cat ID)	FIP Type at Diagnosis	Sex, Age at Diagnosis	Number of Relapses	Day of Relapse*	Original Treatment	First Re-treatment	Second Re-treatment	Third Re-treatment	Outcome**
FeLV− (89)	Ocular	MI, 5 mo	2	3, 244	10 mg/kg 84 days	20 mg/kg 84 days	20 mg/kg 84 days	N/A	Day 778: Remission
FeLV− (125)	Neurologic	FS, 2 mo	2	79, 334	10–15 mg/kg 85 days	15 mg/kg 85 days	20 mg/kg 84 days	N/A	Day 526: Deceased (unrelated trauma)
FeLV− (11)	Neurologic	MN, 36 mo	1	93	10–15 mg/kg 87 days	20 mg/kg 84 days	N/A	N/A	Day 665: Remission
FeLV− (79)	Neurologic	FS, 19 mo	1	448	15 mg/kg 84 days	20 mg/kg 87 days	N/A	N/A	Day 820: Remission
FeLV+ (108)	Neurologic	MN, 18 mo	1	5	12 mg/kg 84 days	20 mg/kg 84 days	N/A	N/A	Day 876: Remission
FeLV+ (133)	Effusive	FS, 2 mo	1	12	6–10 mg/kg 89 days	15 mg/kg 124 days	N/A	N/A	Day 524: Deceased (neoplasia)
FeLV+ (112)	Effusive	MN, 4 mo	1	46	10 mg/kg 87 days	20 mg/kg 84 days	N/A	N/A	Day 275: Deceased (neoplasia)
FeLV+ (37)	Effusive	FI, 7 mo	3	46, 246, 382	10 mg/kg 86 days	15–20 mg/kg 86 days	25 mg/kg 84 days	30 mg/kg 113 days	Day 748: Remission
FeLV+ (17)	Non-effusive	MN, 18 mo	1	48	10 mg/kg 84 days	15 mg/kg 44 days	N/A	N/A	Day 226: Deceased (neoplasia)
FeLV+ (52)	Effusive	MI, 4 mo	1	59	10 mg/kg 84 days	20 mg/kg 98 days	N/A	N/A	Day 523: Deceased (anemia)
FeLV+ (99)	Ocular	MN, 5 mo	1	88	12 mg/kg 84 days	Unknown 84 days	N/A	N/A	Day 368: Deceased (undefined illness)
FeLV+ (77)	Neurologic	MN, 13 mo	1	108	15 mg/kg 95 days	20 mg/kg 84 days	N/A	N/A	Day 627: Deceased (hypertrophic cardiomyopathy)
FeLV+ (62)	Effusive	MN, 13 mo	1	359	12 mg/kg 84 days	20 mg/kg 82 days	N/A	N/A	Day 838: Deceased (undefined illness)
FeLV+ (68)	Effusive	FS, 7 mo	1	375	10 mg/kg 84 days	Unknown 91 days	N/A	N/A	Day 729: Deceased (undefined illness)
FeLV+ (170)	Effusive	FI, 7 mo	1	422	10 mg/kg 85 days	15 mg/kg 91 days	N/A	N/A	Day 765: Remission

MI = male intact, MN = male neutered, FI = female intact, FS = female spayed. *Day of relapse is the number of days until relapse from the END of the first treatment. **Outcome day is the number of days until final follow-up or death from the START of the first treatment. ***Doses are for subcutaneous injections.

## 4 Discussion

In this retrospective study, cats with and without FeLV co-infection had equivalent survival rates throughout the treatment and observation periods (74%). Mortality was highest during the first week of treatment, with 55% of all deaths occurring during that time. After the first critical week, 86% of the remaining cats survived through the observation period. These survival rates were consistent with previous reports in which most exceeded 75% (range 55–100%) (1, 4–19).

The FeLV-positive and FeLV-negative groups were similar at the beginning of treatment with the exception that cats in the FeLV-positive group were on average older (median 10 months) than cats in the FeLV-negative group (median 4 months). FeLV-positive cats were also found to be older at the time of intake to the shelter. The age difference may be attributed to selection bias related to intake policies at the animal shelter, which selectively took in FeLV-positive cats from other shelters. FeLV-positive cats that were younger at the time of diagnosis showed improved survival, whereas there was no difference in survival based on age in the FeLV-negative group. This finding could indicate the importance of early FIP diagnosis and treatment in the survival of FeLV-positive cats, but further studies with equal age distribution would be required to conclude this.

Hematological and biochemical abnormalities classically associated with FIP were common in both groups, including decreased hematocrit, albumin, and albumin to globulin ratio and increased globulin, neutrophils, and neutrophil to lymphocyte ratio. These abnormalities resolved during treatment in responding cats as previously reported (4–6, 8, 13, 14, 18, 19). The response to treatment and normalization of blood tests mirrored that of previous studies in which diagnosis was supported by more advanced diagnostics (5–8, 15). These findings provide support for the original diagnosis of FIP for cats in this study and the use of a spectrum of care approach when resource limitations or access to advanced diagnostics might delay or prevent life-saving treatment.

The most striking difference between the groups occurred following the post-treatment observation period. While both groups had similar survival rates for the first 6 months, their outcomes diverged thereafter. FeLV-positive cats experienced progressive mortality following the observation period with a median survival of only 1.5 years, whereas FeLV-negative cats had no further FIP-related mortality and did not reach median survival during up to 4 years follow-up. This pattern is consistent with the progressive mortality commonly reported for FeLV-infected cats with median survival rates of 0.9–2.4 years following diagnosis (22, 23, 25, 26). In using the spectrum of care approach, this shelter did not routinely perform quantitative FeLV PCR (qPCR) to confirm infection or proviral load following a positive FeLV antigen test result as results would not affect the shelter’s treatment decisions. In a previous study at this same shelter, we showed that of 127 cats initially FeLV-positive by SNAP and retested with SNAP and PCR monthly for 6 months, 119 (94%) had at least one positive qPCR test. Thus, in this population enriched for FeLV infections via the importation of FeLV-positive cats from other shelters for its specialty FeLV adoption program, a single positive SNAP test had a higher probability of accuracy than expected for a population with low prevalence of infection (22).

GS and other FIP antivirals have not been available long enough to observe treated cats over their natural lifespan, and many reports of GS therapy are limited to the early treatment and observation periods, with follow-up typically lasting less than a year. As a result, it remains unclear whether normalization of clinical signs and laboratory test results represents clinical remission versus true cure from viral infection and whether recurrences of clinical signs indicate relapse of a previously unresolved infection versus reinfection following a previous cure. In this study, cats surviving the initial course of treatment and co-infected with FeLV had nearly twice the rate of relapse (13%) as FeLV-negative cats (8%), but this difference did not reach statistical significance. These cats were administered additional rounds of treatment at higher doses to achieve a second or third remission. This relapse rate is consistent with previous reports of GS-treated cats of 0–37% (4–6, 8, 9, 11, 13–15, 17–19).

It has long been recognized that cats with neurological and/or ocular disease have higher FIP treatment failure and relapse rates. This is not surprising as barriers to these anatomically privileged sites frequently inhibit drug penetration (7). GS concentrations were reported to be less than 25% in aqueous humor (22–33% of plasma) and CSF (7–21% of plasma) in 2 cats tested (3). Based on the cumulative reports to date, it appears that cats with neurological or ocular disease can respond to GS treatment, but the optimal level and duration of dose intensification, if any, required to overcome the blood–brain and blood-eye barriers to GS penetration are not yet known.

The amount of GS administered in published reports has trended upwards in both dose and duration since the original reports of its efficacy against FIP in 2018. The first reported success in treating cats with GS came from dosing 10 experimentally infected cats at 2–5 mg/kg daily for 14 days, with a second treatment course for 2 cats (20%) that relapsed (3). The first prospective clinical trial of 31 naturally infected cats used 2–4 mg/kg SC daily for 12 weeks; 8 of 26 cats (31%) that completed treatment in that study relapsed and were retreated (4). Since then, retrospective studies of social network-driven treatments and prospective clinical trials have reported dosages of 2–25 mg/kg once or twice a day, with the highest doses reserved for cats in critical condition, with neurological or ocular disease, and for retreatment of cats suffering from relapse of FIP (5–11, 13, 15–17, 19). Despite initial concerns about the possible reduced bioavailability of GS administered by the oral route, recent pharmacological studies and clinical trial outcomes support oral administration (6, 11, 13, 14, 18, 32). Most reports have generally adhered to a treatment period of 84 days, followed by monitoring clinical signs and laboratory values for another 84 days for a total of approximately 6 months. Some studies were confounded by discrepancies between the labeled concentration of GS and subsequent post-publication analytical determination that actual concentrations were commonly higher or lower than claimed on the label (12, 33). Our study attempted to avoid such discrepancies by using single-source compounded GS analyzed for purity and concentration.

The current study included cats treated since 2020 when global case experience was coming primarily from cat owners participating in online discussion groups and not from veterinary professionals. Peer-to-peer treatment advice was based on accumulating anecdotal reports rather than on structured evidence-based medicine. Belatedly, it was determined that many sources of unregulated GS contained substantially higher or lower drug concentrations than indicated on the label, further complicating the identification of best treatment practices and interpretation of published studies reporting on treatment of cats with unregulated GS supplies (12, 32). It was within this context of uncertainty that the shelter’s veterinarians developed treatment plans for cats in this study. Starting dosages ranged from 10 to 30 mg/kg once or twice a day for 84 days or more based on their assessment of each cat’s condition and progress.

Most recently, a prospective clinical trial in 40 FeLV-negative cats with effusive FIP without neurological or ocular signs compared treatment at 15 mg/kg PO daily for 42 days versus 84 days (18). Nineteen cats (95%) in each group survived at least 168 days and were considered cured, prompting the authors to recommend this abbreviated high-dose protocol for treatment of FIP without neurological or ocular disease. Taken together, the steadily intensifying GS dosing regimens since the original discovery appear to have improved survival and cure rates and reduced relapse rates.

In this study, all candidates for GS treatment were included in the survival analysis, including cats that were critically ill, FeLV-positive, or affected by neurological and/or ocular disease. Cats were followed for a minimum of 1 year and up to 4 years by the time of publication, providing ample time for relapse and mortality events to be recorded. However, even with these high-risk enrollees, survival through the observation period was 74% for both groups, and the relapse rate was only 11%.

This study had several limitations, mostly related to the retrospective nature of the data collection from medical records and selection bias related to the shelter’s cat admission policies. The shelter’s spectrum of care approach to diagnosis was based on the shelter veterinarian’s assessment of patient signalment, history, physical examination, and compatible laboratory findings, congruent with recommendations from the European Advisory Board on Cat Diseases and other published reports when more advanced diagnostics are unavailable (1, 2, 31). The diagnosis of FeLV infection was based on the shelter’s protocol for a single antigen test. Necropsies on deceased cats were not performed as the cats lived with foster and adoptive homes. Therefore, it is possible the diagnosis of FIP and/or FeLV was incorrect in some cases. However, specific laboratory abnormalities identified at the time of diagnosis and their normalization during treatment are classical findings described for FIP that lend credence to its correct diagnosis. Similarly, the shelter’s previous confirmation of 94% of positive FeLV antigen results in its population by PCR suggests a sound testing program (22). The progressive mortality observed only in the FeLV-positive group is also characteristic of FeLV infection. The GS treatment protocol allowed for modification of medication dose, frequency, and route of administration based on subjective clinical assessment of condition and laboratory results at the time of diagnosis and at recheck examinations, creating variability in treatments between cats, which may have impacted outcomes in some cases. For example, FeLV-positive cats were started at a higher dose of GS, potentially leading to a more favorable outcome than if they were treated at the same dose as the FeLV-negative cats.

## 5 Conclusion

The animal shelter in this report used a spectrum of care approach to diagnosis of FIP. This preserved resources for treatment, which successfully induced resolution of clinical and laboratory abnormalities in 74% of cats, even those diagnosed with FeLV infection. However, while FeLV-positive cats responded equally well to initial FIP treatment, they still experienced the overall shorter survival times that have long been associated with FeLV infection. GS has recently become widely available in the US through compounding pharmacies, which will facilitate more shelters, private practices, insurance companies, and academic institutions in openly supporting treatment of FIP, exchanging information, and conducting prospective clinical trials with well-characterized medication. Future studies are needed to identify optimal dosing regimens and treatment intervals tailored to the risk factors and conditions of cats at the time of diagnosis and the role of FeLV proviral load on survival of co-infected cats treated for FIP. Studies specific to shelter issues are needed to determine best practices for vaccination, parasite treatment, and spay/neuter surgery timing in cats undergoing FIP treatment and the risk of FIP developing in littermates of affected kittens. And importantly, more research is also needed in animal shelters to identify risk factors for FCoV transmission, high viral replication rates, and subsequent development of FIP, with goals to develop shelter operations and alternatives to shelter intake that reduce occurrence of shelter-acquired FIP and the need for its treatment.

## Data Availability

The raw data supporting the conclusions of this article will be made available by the authors, without undue reservation.
